# Effect of Reduction of Redox Modifications of Cys-Residues in the Na,K-ATPase α1-Subunit on Its Activity

**DOI:** 10.3390/biom7010018

**Published:** 2017-02-21

**Authors:** Elena A. Dergousova, Irina Yu. Petrushanko, Elizaveta A. Klimanova, Vladimir A. Mitkevich, Rustam H. Ziganshin, Olga D. Lopina, Alexander A. Makarov

**Affiliations:** 1 Engelhardt Institute of Molecular Biology, Russian Academy of Sciences, Vavilov St, 32, Moscow 119991, Russia; dergousova90@list.ru (E.A.D.); irina-pva@mail.ru (I.Yu.P.); klimanova.ea@yandex.ru (E.A.K.); mitkevich@gmail.com (V.A.M.); aamakarov@eimb.ru (A.A.M.); 2 Faculty of Biology, Lomonosov Moscow State University, Leninskie Gory, 1/12, Moscow 119234, Russia; 3 Shemyakin-Ovchinnikov Institute of Bioorganic Chemistry, Russian Academy of Sciences, Miklukho-Maklaya str., 16/10, Moscow 117997, Russia; ziganshin@mail.ru

**Keywords:** Na,K-ATPase, α1-subunit, cysteine residues, oxidation, *S*-glutathionylation, *S*-nitrosylation

## Abstract

Sodium-potassium adenosine triphosphatase (Na,K-ATPase) creates a gradient of sodium and potassium ions necessary for the viability of animal cells, and it is extremely sensitive to intracellular redox status. Earlier we found that regulatory glutathionylation determines Na,K-ATPase redox sensitivity but the role of basal glutathionylation and other redox modifications of cysteine residues is not clear. The purpose of this study was to detect oxidized, nitrosylated, or glutathionylated cysteine residues in Na,K-ATPase, evaluate the possibility of removing these modifications and assess their influence on the enzyme activity. To this aim, we have detected such modifications in the Na,K-ATPase α1-subunit purified from duck salt glands and tried to eliminate them by chemical reducing agents and the glutaredoxin1/glutathione reductase enzyme system. Detection of cysteine modifications was performed using mass spectrometry and Western blot analysis. We have found that purified Na,K-ATPase α1-subunit contains glutathionylated, nitrosylated, and oxidized cysteines. Chemical reducing agents partially eliminate these modifications that leads to the slight increase of the enzyme activity. Enzyme system glutaredoxin/glutathione reductase, unlike chemical reducing agents, produces significant increase of the enzyme activity. At the same time, the enzyme system deglutathionylates native Na,K-ATPase to a lesser degree than chemical reducing agents. This suggests that the enzymatic reducing system glutaredoxin/glutathione reductase specifically affects glutathionylation of the regulatory cysteine residues of Na,K-ATPase α1-subunit.

## 1. Introduction

Sodium-potassium adenosine triphosphatase (Na,K-ATPase) is an enzyme that transports Na^+^ and K^+^ through the plasma membrane against the electrochemical gradient. The transport of cations is coupled with hydrolysis of adenosine triphosphate (ATP) to adenosine diphosphate (ADP) and inorganic phosphate (P_i_ ), which supplies the needed energy. The enzyme is composed of two subunits (catalytic α-subunit and regulatory β-subunit) having molecular masses of 110 and 65 kDa, respectively. Four isoforms of the Na,K-ATPase α-subunit and three isoforms of β-subunit are now known [1]. The isoforms of the two subunits can be combined in different ways creating various heterodimers. The α1β1-isoenzyme is a ‘housekeeping’ enzyme located almost in all animal tissues. The α1β1 enzyme is the only isoenzyme found in tissues providing sodium excretion (mammalian kidney, salt glands of birds). The distribution of other isoforms is tissue-specific.

The polypeptide chain of the Na,K-ATPase α-subunit crosses the membrane 10 times, and its transmembrane segments form a channel with gates for ion transport [2]. Cytoplasmic part of the polypeptide chain is assembled in three cytoplasmic domains. Two of these (nucleotide binding and phosphorylated domains) participate in the formation of the enzyme active site. The Na,K-ATPase α-subunit is required for transport of the enzyme to the membrane, and it affects K^+^ transport [3].

The α1-subunit contains 23 cysteine residues, 15 of them facing the cytosol. There are no disulfide bonds between cysteine residues of the α-subunit [4,5]. Changing cysteine residues to alanine in the α-subunit is not critical for enzyme activity [6,7], so the role of the cysteines is not clear. However, Na,K-ATPase is extremely sensitive to intracellular redox status [4,8,9,10,11,12,13]. This suggests that redox modification of cysteines may play an important role in the regulation of Na,K-ATPase.

We found earlier that Na,K-ATPase from various tissues (rabbit kidney, rat heart, duck salt glands) has a significantly *S*-glutathionylated α1-subunit [8,9,10]. The level of the α1-subunit glutathionylation depends on cellular redox status and increases under hypoxia [8,10]. In the Na,K-ATPase α1-subunit from duck salt glands, up to 12 of the 15 cysteine residues facing the cytosol can be *S*-glutathionylated [8]. Treatment of Na,K-ATPase from this tissue with oxidized glutathione in vitro resulted in the glutathionylation of Cys-244, -454, -458, and -459 of the α1-subunit, leading to complete inhibition of the enzyme activity. After glutathione binding to these cysteines, the enzyme was unable to interact with adenine nucleotides [8]; apparently glutathione bound to three cysteine residues (Cys-454, -458, and -459) near the ATP-binding site can prevent ATP binding [8]. Because adenine nucleotides can prevent the enzyme glutathionylation and ATP is most effective in this respect [8,9], depletion of ATP will induce glutathionylation. It was shown that the only free SH-group of β-subunit could bind glutathione, which decreases the enzyme activity by approximately 20% [14]. Thus, glutathionylation of some cysteines of Na,K-ATPase subunits is involved in the regulation of its activity.

In addition to four regulatory cysteine residues of Na,K-ATPase α1-subunit that are glutathionylated after treatment with oxidized glutathione, many other cysteines are in the glutathionylated form in purified enzyme. The functional significance of their *S*-glutathionylation remains unclear. We suggested that glutathionylation of some cysteines of the Na,K-ATPase α-subunit takes place cotranslationally and is important for protein folding [5]. It was shown earlier that hypoxic rat heart cysteine residues of Na,K-ATPase can be also *S*-nitrosylated, and that in hypoxic rat heart there is cross talk between *S*-nitrosylation and *S*-glutathionylation of the Na,K-ATPase α1-subunit [15]. Oxidation of SH-groups to -SOH in proteins can proceed to their glutathionylation or nitrosylation, but irreversibly oxidized cysteines (to cys-SO_2_H and cys-SO_3_H) are now considered as markers for protein degradation [4].

To better understand the role of various redox modifications of cysteine residues of the Na,K-ATPase α-subunit in its function, we have studied effects of different reducing agents and the enzymatic reducing system glutaredoxin/glutathione reductase on the modified cysteine residues of Na,K-ATPase and on the activity of the enzyme.

## 2. Results

**Figure 1:**
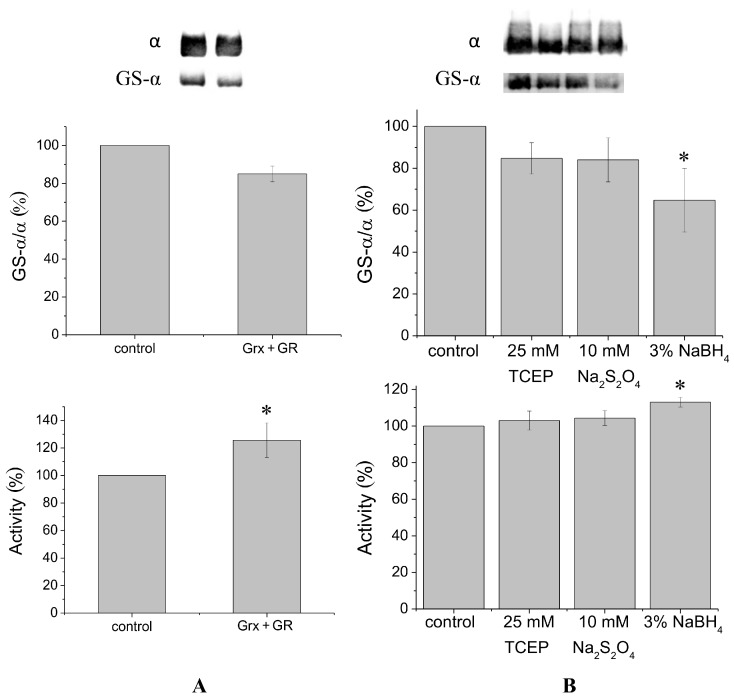
Deglutathionylation of the Na,K-ATPase α1-subunit in the absence of denaturing reagents. (**A**) Deglutathionylation using the coupled enzyme system glutaredoxin (20.6 µg/mL) and glutathione reductase in the presence of 0.5 mM glutathione and 200 µM nicotinamide adenine dinucleotide phosphate (NADPH). Samples were incubated for 30 min at 37 °C. Control samples (taken as 100%) were incubated under the same conditions without reduced glutathione and the enzymes. The upper panel presents results of immunoblotting with staining by antibodies against bound glutathione and against Na,K-ATPase α1-subunit. The lower panel shows change in enzyme activity (number of experiments: *N* = 3); (**B**) Deglutathionylation using chemical reducing agents: 25 mM tris(2-carboxyethyl)-phosphine (TCEP), 10 mM Na_2_S_2_O_4_, and 3% NaBH_4_. The incubation time was 30 min at 37 °C. The upper panel depicts immunoblotting with staining by antibodies against bound glutathione and against the α1-subunit. The results were normalized to the α1-subunit content. Lower panel: change in Na,K-ATPase activity after treatment by reducing agents (*N* = 4). Data are presented as mean ± standard deviation (SD) of independent experiments. * *p* < 0.05.

It is known that glutaredoxin combined with glutathione reductase in the presence of their substrates glutathione and nicotinamide adenine dinucleotide phosphate (NADPH) can catalyze protein deglutathionylation [4,8]. Incubation of duck salt gland Na,K-ATPase in vitro with glutaredoxin/glutathione reductase for 30–60 min results in a small decrease in the level of Na,K-ATPase α1-subunit glutathionylation detected using antibodies against glutathione bound to proteins (Figure 1A, Supplementary Figure S2). Although deglutathionylation of the α1-subunit using glutaredoxin/glutathione reductase does not exceed ~20%, it increases Na,K-ATPase activity by ~30% (Figure 1A).

To deglutathionylate natively glutathionylated Na,K-ATPase α1-subunit, we also used chemical reducing agents tris(2-carboxyethyl)-phosphine (TCEP), sodium dithionite, and sodium borohydride, that have redox potentials of −0.29, −0.416, and −1.24 V, respectively. We previously found concentrations of these reagents that provide the maximal effect (data not shown). Treatment of Na,K-ATPase by TCEP, sodium dithionite, or sodium borohydride for 30 min at 37 °C decreased glutathionylation level of the α1-subunit by 15%–35% (Figure 1B, Supplementary Figure S3). Sodium borohydride, having the lowest redox potential, provided the maximal effect. Dithiothreitol and β-mercaptoethanol, which have redox potentials comparable with TCEP (−0.33 and −0.26 V, respectively) decreased the level of glutathionylation almost as TCEP (by ~12%, see Supplementary Figure S6). In contrast to enzymatic deglutathionylation, treatment of Na,K-ATPase with reducing agents was accompanied by a slight increase in Na,K-ATPase activity (Figure 1B). The maximal increase (less than 20%) was observed after treating the enzyme with sodium borohydride (Figure 1B).

**Figure 2:**
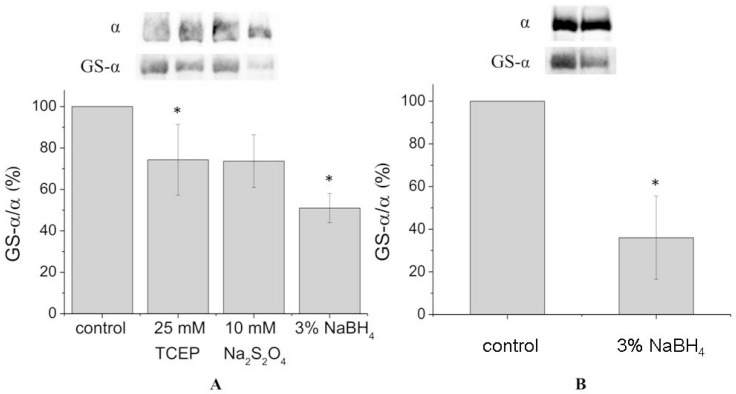
Deglutathionylation of α1-subunit under denaturing conditions. (**A**) Deglutathionylation of the duck salt gland α1-subunit (after treatment with 25 mM TCEP, 10 mM Na_2_S_2_O_4_, or 3% NaBH_4_) in the presence of 8 M urea and 8% sodium dodecyl sulphate (SDS) for 30 min at 37 °C. Results of immunoblotting after incubation of the enzyme with antibodies against bound glutathione and the α1-subunit are presented. The results were normalized to the α1-subunit content (number of experiments: *N* = 3); (**B**) Microsomes of duck salt glands after treatment with 8 M urea and 8% SDS for 30 min at 37 °C. Results of immunoblotting after incubation of the sample with antibodies against glutathione and the α1-subunit are presented. The results were normalized to the α1-subunit content (number of experiments: *N* = 3). Data are presented as means ± SD of independent experiments. * *p* < 0.05.

In order to achieve complete deglutathionylation of the α1-subunit, we treated the enzyme with chemical reducing agents under denaturing conditions (in the presence of 8% sodium dodecyl sulfate (SDS) and 8 M urea at 37 °C). However, even under these conditions deglutathionylation of the α1-subunit was partial, not exceeding 50% (Figure 2A, Supplementary Figures S4 and S7). For the microsomal fraction, deglutathionylation of the α1-subunit reached 65% (Figure 2B, Supplementary Figure S5).

To check for the presence of glutathione bound to the α1-subunit after its treatment with reducing agents, we performed mass spectrometry analysis of enzyme preparation that was incubated with or without sodium borohydride under denaturing conditions (Table 1 and Supplementary Tables S1 and S2, sequence coverage was 69.0% and 69.9% correspondingly). Supplementary Tables contain information about tryptic peptides of α1-subunit with different redox modifications of cysteine residues in the control sample (Supplement Table S1) and sample treated by sodium borohydride (Supplement Table S2) under denaturing conditions. Table 1 shows the numbers of different tryptic peptides (including peptides with sequences having different numbers of trypsin miscleavage sites) with a determined redox modification of the given cysteine residue under control conditions (Control) and after treatment with sodium borohydride (Reduced). Firstly, we should note that patterns of tryptic cleavage of the α1-subunit polypeptide chain before and after enzyme treatment by sodium borohydride were different. In the control preparation, we found 28 different tryptic fragments of the α1-subunit polypeptide chain containing 13 cysteine residues (Supplementary Table S1). After the reduction of cysteine residues of Na,K-ATPase under denaturing conditions, we ascertained 26 tryptic fragments of the α-subunit containing 13 cysteine residues (Supplementary Table S2). After the reduction of the denatured protein, we found only five peptides containing *S*-glutathionylated cysteine residues (Cys-454, -458, -459, -513, and -658).

Three of the cysteine residues of the α1-subunit of Na,K-ATPase (Cys-140, -206, and -351) after reduction of the denatured enzyme lost their bound glutathiones. We also observed a decrease in the number of peptides containing glutathionylated Cys-454, -458, and -459 after the reduction of the denatured protein.

Cys-423 was found in tryptic fragments of native enzyme only in the unmodified form, which is consistent with our results obtained earlier [8]. However, we did not observe peptides with modified Cys-369 although earlier we detected a low intensity peak for the peptide with glutathionylated Cys-369 [8]. Eight cysteine residues of the α1-subunit (Cys-140, -206, -351, -454, -458, -459, -513, and -658) were *S*-glutathionylated. There were no peptides with glutathionylated Cys-244 and Cys-700 (Table 1), which we have detected in other preparations where denaturing conditions did not apply [8]. However, in the analyzed preparation of Na,K-ATPase, these cysteine residues were oxidized, which could prevent their glutathionylation (Table 1). We also detected a peptide with glutathionylated Cys-140, which was not revealed earlier [8]. After reduction under denaturing conditions Cys-140, -206, and -351 lost their bound glutathione. We also observed a decrease in the number of peptides containing glutathionylated Cys-454, -458, and -459 (Table 1).

**Figure 3:**
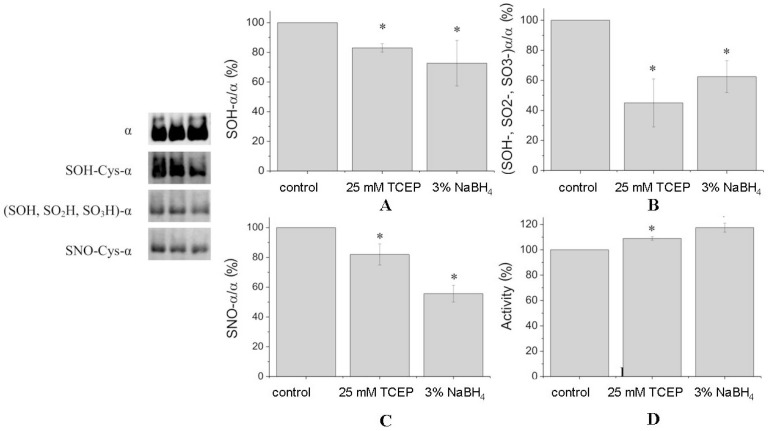
Chemical modifications of the α1-subunit (**A**–**C**) and changes in Na,K-ATPase activity (**D**) after treatment of the enzyme by TCEP (25 mM) and sodium borohydride (3%). The Na,K-ATPase was incubated with or without reducing agent for 30 min at 37 °C. The left panel depicts immunoblotting with staining by antibodies against α1-subunit by antibodies against sulfenic acid (Cys-SOH), against all oxidized SH-groups (Cys-SOH, Cys-SO_2_H, Cys-SO_3_H), and against nitrosylated cysteine thiols (Cys-SNO). Diagrams representing densitometric analysis of immunoblots: (**A**) α1-subunit stained using antibodies against sulfenic acid (Cys-SOH); (**B**) α1-subunit stained using antibodies against all oxidized SH-groups (Cys-SOH, Cys-SO_2_H, Cys-SO_3_H); (**C**) α1-subunit stained using antibodies against *S*-nitrosylated cysteine thiols (Cys-SNO). The bars are normalized to the α1-subunit content stained by antibodies against α1-subunit after stripping (number of experiments: *N* = 3). Data are presented as means ± SD of independent experiments. * *p* < 0.05.

Four cysteine residues of the α1-subunit (Cys-351, -454, -458, -459) were found in nitrosylated forms (Table 1). Nitrosylated Cys-700 was revealed only in the preparation after reduction. This may be due to the fact that the pattern of tryptic cleavage after reduction was changed and we did not detect a peptide corresponding to nitrosylated Cys-700 in the reduced preparation. After treatment of denaturated protein by reducing agents, the number of nitrosylation of cysteine residues has not changed but the total number of nitrosylated peptides decreased. Treatment of undenatured Na,K-ATPase by TCEP and NaBH_4_ decreased the amount of Cys-SNO by 20% to 57%, correspondingly (Figure 3C).

Some cysteines were oxidized to Cys-SOH and Cys-SO_2_H (Table 1). TCEP and sodium borohydride decreased the amount of Cys-SOH in α1-subunit by 20% to 30% (Figure 3A). However, the level of all oxidized cysteines (Cys-SOH, Cys-SO_2_H, Cys-SO_3_H) of the α1-subunit was decreased by TCEP and sodium borohydride by ~60% and ~40%, respectively (Figure 3B).

It can be seen that many cysteines of the α1-subunit had several different redox modifications. Cys-351 that had all types of redox modifications was found after the reduction only in the unmodified and nitrosylated forms. Peptides including Cys-456, -458, and -459 before and after treatment of protein with reducing agents were found in unmodified forms and with all types of cysteine residue modifications, although the total number of modified peptides was decreased (Table 1).

Effects of reducing agents on all the modifications of cysteine residues studied using antibodies against specific modifications of cysteines were confirmed by mass spectrometry. The data presented in Table 1 demonstrate that the total number of peptides containing all types of redox modifications of cysteines of α1-subunit dropped about two-fold after the reduction of denatured Na,K-ATPase by sodium borohydride.

## 3. Discussion

The Na,K-ATPase α1-subunit from different sources has 23 cysteine residues [4]. Individual replacement of cysteines by alanine or by serine in the α1-subunit resulted in some cases in decrease of the enzyme turnover by 2–4-fold [6]. However, replacement of Cys-242 (Cys-244 in duck α1-subunit) led to the cessation of the enzyme expression. Molecular activity of the cysteine-less (cysteine residues in the α-subunit were substituted with alanines or serines) mutants of Na,K-ATPase and the K_m_ values for activation by ATP were similar to the values characteristic for the wild-type enzyme [7]. However, a number of parameters for cysteine-less enzyme were different: 50% reduction of K_m_ for Na^+^, and 2-fold increase in value for K_m_ for K^+^ were observed. Moreover, the stability of cysteine-less enzyme was decreased significantly. The cysteine-less enzyme has higher content in endoplasmic reticulum and Golgi apparatus in comparison with the wild-type protein, it has lower stability [7]. Thus, cysteines are not critical for function, but some of them appear to be necessary for α1-subunit folding, trafficking, assembly, and stability [7]. Despite the replacement of cysteine residues, enzyme activity does not change [6,7]; however, modification of regulatory cysteine residues by glutathione can result in complete enzyme inhibition [8]. We suggest that cysteines are important for redox-sensitive regulation of Na,K-ATPase. Oxidative modifications of Na,K-ATPase thiols may also be involved in oxygen-inducible regulation of enzyme functions [4,8].

We showed that in addition to regulatory glutathionylation of the α1-subunit (Cys-454, -458, -459, -244) (Supplementary Figure S8), basal glutathionylation of the Na,K-ATPase that cannot be removed in the native protein also exists [5]. Unresolved density in isolated cavities in X-ray structure of pig Na,K-ATPase can be occupied by molecules of glutathione associated with the pairs of cysteine residues 204–242, 367–698, and 452–456 (206–244, 369–700, and 454–458 in the duck α1-subunit, which are located in actuator (A), nucleotide binding (N), and phosphorylated (P) domains respectively (Supplementary Figure S8) [4,5]. According to our previous data [5], cysteine residues 204, 452, and 698 are preferable for basal glutathionylation. It should be noted that each of the domains (A, N, and P) contains basal glutathionylated residue (Supplementary Figure S8). We suggested that basal glutathionylation can prevent the formation of disulphide bridges between the neighboring cysteine residues during folding of α-subunit [5]. Formation of disulfide bridges can increase structure rigidity and prevents conformational lability of the molecule, needed for moving Na,K-ATPase domains in catalytic cycles. We assumed that this basal glutathionylation occurs cotranslationally and ‘remembers’ the cellular redox state that existed during protein biosynthesis. 

According to our data (Figure 1) application of coupled enzyme system glutaredoxin/glutathione reductase unlike chemical reducing agents leads to significant increase of enzyme activity. At the same time the enzyme system glutaredoxin/glutathione reductase deglutathionylates native Na,K-ATPase in a lesser degree than chemical reducing agents, so the enzyme system deglutathionylates only some regulatory thiol groups [8].

We have not been able to fully remove bound glutathiones from the denatured purified α-subunit even by applying borohydride. However, we completely removed glutathionylation in the denatured murine α1-subunit previously [5]. This can be explained by differences in the microenvironment of cysteines in α1-subunits from duck and mouse which can change their susceptibility to glutathionylation. The nearest-neighbor residues to cysteines in the α1-subunit sequence affect the pK_a_ of Cys thiol group, changing its reactivity [4]. Another example of an extra stable disulfide bond, which was not disrupted even at 60 °C in the presence of 10 mM dithiothreitol appears in the double cysteine mutant of subtilisin between Cys-22 and Cys-87 [16].

Taking into account all obtained data, we can suggest the following concerning the function of glutathionylation of cysteine residues of Na,K-ATPase α1-subunit. Short time oxidative stress and short time hypoxia that lead to the increase of GSSG (oxidazed glutathione, hexapeptide) concentrations results in the increase of glutathionylation of regulatory cysteines in the α-subunit (located in the N or A domains, Figure S8) that inhibits the enzyme and prevents ATP exhaustion in the cells [8]. At the same time, prolonged oxidative stress or prolonged hypoxia lead to the glutathionylation of some cysteines that proceeds during protein folding. This increase of basic glutathionylation of Na,K-АТPase α1-subunit that cannot be removed from native protein [5], because the modified residues (located in the A, N, and P domains, Supplementary Figure S8) are inaccessible for reduction agents. We suggest that increase of basic glutathionylation results in the change of protein properties that, in turn, may be important for the adaptation to prolonged oxidative stress. At the same time, the glutathionylation of non-regulatory (but accessible for reducing agents) cysteine residues will protect the enzyme from irreversible oxidation. We have found that, in Na,K-АТPase purified from duck salt glands, there is some glutathionylated cysteine residues reduction of which using enzyme system results in the increase of enzyme activity. We have also found some cysteine residues whose deglutathionylation does not affect enzyme activity. Additionally, we have found some basically glutathionylated cysteine residues that cannot be deglutathionylated under different conditions. Only Cys-423 buried in N domain (Supplementary Figure S8) both previously [8] and now (Table 1) was found in the unmodified form. Therefore, we can place cysteine residues of Na,K-ATPase α1-subunit into three functional groups: regulatory cysteines (responsible for the enzyme activity, their glutathionylation leads to enzyme inhibition), basically glutathionylated residues that are important for protein folding and prolonged adaptation, and normal cysteine residue glutathionylation which does not affect the activity, but protects thiol groups against irreversible oxidation.

Earlier, it was hypothesized that nitrosylation of cysteine residues was important for maintaining high activity of Na,K-ATPase at physiological oxygen levels [11,12,13], and that cross talk existed between glutathionylation and nitrosylation of the Na,K-ATPase catalytic subunit. This was demonstrated previously [15]. If nitric oxide synthases activity is decreased during hypoxia, the level of α1-subunit nitrosylation is also decreased, whereas the level of glutathionylation is increased [8,10]. Both modifications protect cysteine residues from irreversible oxidation to sulfinic and sulfonic acids. However, glutathionylation, in contrast to nitrosylation, leads to enzyme inhibition, which can be reversed when the cell returns to the normal redox state using some enzyme coupled systems such as glutaredoxin/glutathione reductase.

Indeed, we observed not only glutathionylation but also nitrosylation of a number of cysteine residues in the α1-subunit of Na,K-ATPase from duck salt glands. The most nitrosylated cysteine residues are the same cysteine residues that can be glutathionylated, which is consistent the with cross-talk hypothesis. Treatment of Na,K-ATPase with reducing agents decreases the degree of nitrosylation of cysteine residues but does not eliminate nitrosylation completely, as well as glutathionylation. Thiol groups of cysteine residues that are not protected by nitrosylation or by glutathionylation seem to be oxidized to sulfenic, sulfinic, and sulfonic acids.

It should be noted that Cys-454, -458, and -459 (two of them are located in a cavity) lying near the ATP-binding site (Supplementary Figure S8) exist in nitrosylated, glutathionylated, and oxidized forms before and after reduction, and these modifications cannot be elicited by reducing agents. This supports our hypothesis that some of these cysteines are involved in the regulation of Na,K-ATPase activity [8] but the others are not involved in the enzyme activity regulation since they are located in isolated cavities [5]. Being glutathionylated during hypoxia, they inhibit the enzyme, but after in vivo deglutathionylation by the glutaredoxin/glutathione reductase enzyme system under normal conditions, they are nitrosylated to prevent irreversible deep (to -SO_2_H and -SO_3_H) oxidation.

Cysteines of the Na,K-ATPase α1-subunit can be oxidized to different levels. Irreversible oxidation of cysteines of the protein to Cys-SO_3_H and Cys-SO_2_H was proposed as a marker for protein degradation by the cellular machinery [17] whereas oxidation to Cys-SOH is considered now as a posttranslational modification that can be important for enzyme regulation [18]. Treatment of Na,K-ATPase with reducing agents decreases the number of Cys-SOH and Cys-SO_2_H (Table 1)*.* This correlates with data of Western blotting, where the amount of all oxidized forms of cysteines is decreased more significantly than the amount of Cys-SOH (Figure 3).

Thus, using two methods, we have found that a number of cysteine residues of Na,K-ATPase purified from duck salt glands are glutathionylated, nitrosylated, and oxidized to a different degree. Reducing agents decreased the level of all these modifications (but not completely) leading to a slight increase in the enzyme activity. The natural enzyme system glutaredoxin/glutathione reductase that decreased glutathionylation to a lesser extent than the reducing agents produced a greater increase in enzyme activity. This suggests that the enzyme deglutathionylation system specifically affects glutathionylation of the regulatory cysteine residues of Na,K-ATPase α-subunit.

## 4. Materials and Methods 

### 4.1. Purification of Na,K-ATPase from Duck Salt Glands 

Microsomes and Na,K-ATPase were purified from duck salt glands [19]. Glands were kept in liquid nitrogen before use. All solutions used for the purification were saturated with argon. The activity of the purified enzyme was 1600–2000 µmol/mg protein*hour. The total content of Na,K-ATPase α1- and β1-subunits in the preparation was shown by polyacrylamide electrophoresis with Coomassie brilliant blue staining to be at least 92% (Supplementary Figure S1). Na,K-ATPase activity was completely suppressed by 1 mM ouabain.

### 4.2. Protein Concentration

Protein concentration was determined using the method of Lowry et al. [20].

### 4.3. Na,K-ATPase Activity

Na,K-ATPase activity was measured as ATP cleavage in a medium containing (mM) 130 NaCl, 20 KCl, 3 MgCl_2_, 3 ATP, and 30 imidazole, pH 7.4, at 37 °C. Inorganic phosphate concentration was determined using the method of Rathbun and Betlach [21].

### 4.4. Immunoblotting

Modifications of cysteines of Na,K-ATPase α1-subunit were assessed by immunoblotting. The proteins were separated on SDS-PAGE (electrophoresis in polyacrylamide gel in the presence of sodium dodecyl sulphate) according to the Laemmli method [22] with 6% concentrating and 10% running gels (the sample buffer did not contain β-mercaptoethanol). Then proteins were transferred to a nitrocellulose membrane. After the blocking procedure, mouse monoclonal anti-glutathione antibody (Chemicon Millipore, Billerica, MA, USA), anti-SOH antibody (Millipore, Billerica, MA, USA, #07-2139), the antibodies recognizing all forms of cysteine with oxidized thiol group (Enzo Life Sciences, Farmingdale, NY, USA, #ADI-OSA-820), or anti-NO antibody (Alpha Diagnostic International, San Antonio, TX, USA, #NISC11-A) were added. The membranes were then stripped, and mouse monoclonal antibody against Na,K-ATPase α1-subunit clone C464-6 (Upstate Millipore, Billerica, MA, USA) was applied to detect the total amount of α1-subunit, followed by horseradish peroxidase-conjugated secondary antibodies. The membranes were stained using the commercial SuperSignal West Femto Maximum Sensitivity Substrate kit (Thermo Scientific, Rockford, IL, USA), and chemiluminescence was detected using a Bio-Rad ChemiDoc MP instrument (Bio-Rad, Hercules, CA, USA). Densitometric analysis was performed using the Image Lab (Bio-Rad) program (Bio-Rad, Hercules, CA, USA), results were represented as a ratio of modified α1-subunit to the total α1-subunit band intensity (modified-α1)/total α1).

### 4.5. Deglutathionylation of Na,K-ATPase Using the Coupled Enzyme System

Bound glutathione was removed from cysteines of Na,K-ATPase in the presence of glutaredoxin and glutathione reductase as described earlier [23]. The Na,K-ATPase was diluted by solution containing 25 mM MOPS (3-(*N*-morpholino)propanesulfonic acid)/imidazole, 2 mM EDTA (Ethylenediaminetetraacetic acid), pH 7.0, to the final concentration of 0.25 mg/mL. Then reduced glutathione (0.5 mM), glutaredoxin (20.6 µg/mL), glutathione reductase (6 µg/mL), and NADPH (200 µM) were added. The reaction was allowed to proceed for 30 min at 37 °C and then was stopped by addition of cold acetate buffer or sample buffer. After that, the Na,K-ATPase activity and the level of modification of cysteine residues were determined.

### 4.6. Deglutathionylation of Na,K-ATPase by Chemical Reducing Agents

Glutathione was removed using chemical reducing agents: sodium dithionite, Na_2_S_2_O_4_ (10 mM), sodium borohydride, NaBH_4_ (3%), TCEP-HCl (25 mM), β-mercaptoethanol (30 mM), dithiothreitol (10 mM). Na,K-ATPase was diluted with solution containing 0.25 M sucrose, 1 mM EDTA, and 25 mM Tris-HCl (pH 7.5) to the concentration of 0.5 mg/mL. The protein was incubated with the reducing agent for 30 min at 37 °C, then aliquots of protein were taken to measure Na,K-ATPase activity. The remaining protein was subjected to dialysis against solution containing 25 mM Tris-HCl and 1 mM EDTA (pH 7.5) for 4 h or was diluted 50-fold by the buffer. Then the level of modification of cysteine residues was measured by immunoblotting or mass spectrometry. Some experiments were done with denaturing agents (8% SDS and 8 M urea).

### 4.7. Mass Spectrometry

Modified cysteine residues of Na,K-ATPase α1-subunit (glutathionylated, oxidized, and nitrosylated) were identified using MALDI-TOF MS (matrix assisted laser desorption ionization-time of flight mass spectrometry). After SDS-PAGE, the band corresponding to Na,K-ATPase α1-subunit was excised from the gel and subjected to in-gel digestion by trypsin [8]. MALDI-TOF MS analysis of the resulting peptide fragments was performed using an Ultraflex II TOF/TOF mass spectrometer (Bruker Daltonics, Bremen, Germany) [8]. The MS data were processed using Bruker Daltonics Flex Analysis 2.4 software (Bruker Daltonics), and the accuracy of mass determination of the peptides was fixed to 100 ppm. The mass spectrometry (MS) data was correlated with the protein sequence using Bruker Daltonics BioTools 3.0 software (Bruker Daltonics).

### 4.8. Statistical Analysis

Values are shown as means and standard deviations. Student’s *t* test was applied, and the difference was considered significant at *p* < 0.05.

## Figures and Tables

**Table 1:** Number of peptides (including sequences with different number of trypsin miscleavage sites) with redox-modified cysteine residues (-SG, glutathionylated Cys; -SNO, nitrosylated Cys; -SOH and -SO_2_H, oxidized Cys) revealed by matrix assisted laser desorption ionization-time of flight mass spectrometry (MALDI-TOF-MS) analysis under control conditions (Control) and after treatment with sodium borohydride (Reduced) under denaturing conditions.

Cysteine No.	-SG		-SNO		-SOH		-SO_2_H	
	Control	Reduced	Control	Reduced	Control	Reduced	Control	Reduced
140	1	-	-	-	-	-	-	-
206	1	-	-	-	-	-	-	-
244	-	-	-	-	1	-	1	1
351	1	-	2	1	1	-	3	-
369	-	-	-	-	-	-	-	-
423	-	-	-	-	-	-	-	-
454, 458, 459	5	2	8	3	6	2	12	6
513	1	2	-	-	-	-	-	-
601	-	-	-	-	2	2	1	2
658	1	1	-	-	-	-	1	1
700	-	-	-	1	1	2	2	1
Total	9	5	10	6	11	6	20	11

## Supplementary Materials

The following are available online at www.mdpi.com/2218-273X/7/1/18/S1, Table S1: Cys-containing peptides (including sequences with different number of trypsin miscleavage sites) detected in MALDI-TOF MS spectra of trypsinized Na,K-ATPase α1-subunit, Table S2: Cys-containing peptides (including sequences with different number of trypsin miscleavage sites) detected in MALDI-TOF MS spectra of trypsinized Na,K-ATPase α1-subunit after reduction with sodium borohydride, Figure S1: Coomassie-stained SDS-PAGE characterization of Na,K-ATPase preparation isolated from duck salt glands, Figure S2: Deglutathionylation of the Na,K-ATPase α-subunit using the coupled enzyme system glutaredoxin (20.6 μg/mL) and glutathione reductase in the presence of 0.5 mM glutathione and 200 μM NADPH (full blot), Figure S3: Deglutathionylation using chemical reducing agents: 25 mM TCEP, 10 mM Na_2_S_2_O_4_ and 3% NaBH_4_ (full blot), Figure S4: Deglutathionylation of duck salt gland α-subunit (after treatment with 25 mM TCEP, 10 mM Na_2_S_2_O_4_, or 3% NaBH_4_) in the presence of 8 M urea and 8% SDS for 30 min at 37 °C (full blot), Figure S5: Microsomes of duck salt glands after treatment with 3% NaBH_4_ in the presence of 8 M urea and 8% SDS for 30 min at 37 °C (full blot), Figure S6: Deglutathionylation of the Na,K-ATPase α-subunit using chemical reducing agents: 10 mM DTT (dithiothreitol) and 30 mM β-ME (β-mercaptoethanol), Figure S7: Deglutathionylation of the Na,K-ATPase α-subunit using chemical reducing agents 10 mM DTT and 30 mM β-ME in the presence of 8 M urea and 8% SDS for 30 min at 37 °C, Figure S8: Cartoon representation of the Na,K-ATPase α1-subunit model in two projections, built on the basis of 2.8 Å structure of the porcine α1-subunit (Protein Data Bank code: 3wgu).

## References

[1] Lingrel J., Moseley A., Dostanic I., Cougnon M., He S., James P., Woo A., O’Connor K., Neumann J. (2003). Functional Roles of the α Isoforms of the Na,K-ATPase. Ann. N. Y. Acad. Sci..

[2] Kaplan J.H. (2002). Biochemistry of Na,K-ATPase. Annu. Rev. Biochem..

[3] Kirley T.L. (1989). Determination of three disulfide bonds and one free sulfhydryl in the *β* subunit of (Na,K)-ATPase. J. Biol. Chem..

[4] Bogdanova A., Petrushanko I.Yu., Hernansanz-Agustín P., Martínez-Ruiz A. (2016). ”Oxygen Sensing” by Na,K-ATPase: These Miraculous Thiols. Front. Physiol..

[5] Mitkevich V.A., Petrushanko I.Y., Poluektov Y.M., Burnysheva K.M., Lakunina V.A., Anashkina A.A., Makarov A.A. (2003). Basal glutathionylation of Na,K-ATPase α-subunit depends on redox status of cells during the enzyme biosynthesis. Oxid. Med. Cell. Longev..

[6] Shi H.G., Mikhaylova L., Zichittella A.E., Arguello J.M. (2000). Functional role of cysteine residues in the (Na,K)-ATPase α-subunit. Biochim. Biophys. Acta.

[7] Hu Y.K., Eisses J.F., Kaplan J.H. (2000). Expression of an active Na,K-ATPase with an α-subunit lacking all twenty-three native cysteine residues. J. Biol. Chem..

[8] Petrushanko I.Y., Yakushev S., Mitkevich V.A., Kamanina Y.V., Ziganshin R.H., Meng X., Anashkina A.A., Makhro A., Lopina O.D., Gassmann M. (2012). *S*-glutathionylation of the Na,K-ATPase catalytic α-subunit is a determinant of the enzyme redox sensitivity. J. Biol. Chem..

[9] Xianyu M., Petrushanko I.Yu., Klimanova E.A., Dergousova E.A., Lopina O.D. (2014). Glutathionylation of the alpha-subunit of Na,K-ATPase from rat heart by oxidized glutathione inhibits the enzyme. Biochemistry.

[10] Petrushanko I.Yu., Simonenko O.V., Burnysheva K.M., Klimanova E.A., Dergousova E.A., Lopina O.D., Makarov A.A. (2015). The ability of cells to adapt to low-oxygen conditions is associated with glutathionylation of Na,K-ATPase. Mol. Biol..

[11] Bogdanova A., Petrushanko I., Boldyrev A., Gassmann M. (2006). Oxygen- and redox-induced regulation of the Na/K-ATPase. Curr. Enzyme Inhib..

[12] Petrushanko I.Yu., Bogdanov N.B., Lapina N., Boldyrev A.A., Gassmann M., Bogdanova A. (2007). Oxygen-induced regulation of Na/K-ATPase in cerebellar granule cells. J. Gen. Physiol..

[13] Petrushanko I., Bogdanov N., Bulygina E., Grenacher B., Leinsoo T., Boldyrev A., Gassmann M., Bogdanova A. (2006). Na-K-ATPase in rat cerebellar granule cells is redox sensitive. Am. J. Physiol. Regul. Integr. Comp. Physiol..

[14] Figtree G.A., Liu C.C., Bibert S., Hamilton E.J., Garcia A., White C.N., Chia K.K., Cornelius F., Geering K., Rasmussen H.H. (2009). Reversible Oxidative Modification. A Key Mechanism of Na^+^-K^+^ Pump Regulation. Circ. Res..

[15] Yakushev S., Band M., van Patot M.C.T., Gassmann M., Avivi A., Bogdanova A. (2012). Cross-talk between *S*-nitrosylation and *S*-glutathionylation in control of Na,K-ATPase regulation in hypoxic heart. Am. J. Physiol. Heart Circ. Physiol..

[16] Wells J.A., Powers D.B. (1986). In Vivo Formation and Stability of Engineered Disulfide Bonds in Subtilisin. J. Biol. Chem..

[17] Davies M.J. (2016). Protein oxidation and peroxidation. Biochem. J..

[18] Yan Y., Shapiro A.P., Haller S., Katragadda V., Liu L., Tian J., Basrur V., Malhotra D., Xie Z.J., Abraham N.G. (2013). Involvement of reactive oxygen species in a feed-forward mechanism of Na/K-ATPase-mediated signaling transduction. J. Biol. Chem..

[19] Smith T.W. (1988). Purification of Na^+^,K^+^-ATPase from the supraorbital salt gland of the duck. Methods Enzymol..

[20] Lowry O.H., Rosebrough N.J., Farr A.L., Randall R.J. (1951). Protein Measurement with the Folin Phenol Reagent. J. Biol. Chem..

[21] Rathbun W.B., Betlach M.V. (1969). Estimation of enzymically produced orthophosphate in the presence of cysteine and adenosine triphosphate. Anal. Biochem..

[22] Laemmli U.K. (1970). Cleavage of structural proteins during the assembly of the head of bacteriophage T4. Nature.

[23] Duppatla V., Gjorgjevikj M., Schmitz W., Kottmair M., Mueller T.D., Sebald W. (2012). Enzymatic deglutathionylation to generate interleukin-4 cysteine muteins with free thiol. Bioconjug. Chem..

