# Supplementary material for: Effect of Reduction of Redox Modifications of Cys-Residues in the Na,K-ATPase α1-Subunit on Its Activity
Source: Biomolecules. 2017 Feb 21;7(1):18. doi: 10.3390/biom7010018 (PMC5372730; doi:10.3390/biom7010018)
Supplement: Supplementary file 1 [file biomolecules-07-00018-s001.pdf]

Supplementary Materials: Effect of Reduction of Redox Modifications of Cys-Residues in the Na,K-ATPase α1-Subunit on Its Activity

Elena A. Dergousova, Irina Yu. Petrushanko, Elizaveta A. Klimanova, Vladimir A. Mitkevich, Rustam H. Ziganshin, Olga D. Lopina, and Alexander A. Makarov

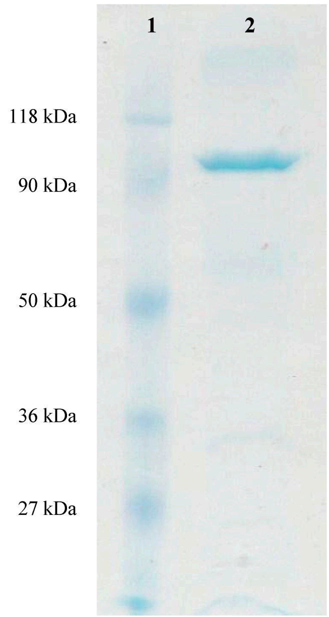

Figure S1. Coomassie-stained SDS-PAGE (polyacrylamide gel electrophoresis in the presence of sodium dodecyl sulfate) characterization of sodium-potassium adenosine triphosphatase (Na,K-ATPase) preparation isolated from duck salt glands. 1—molecular weight standards; 2—purified Na,K-ATPase.

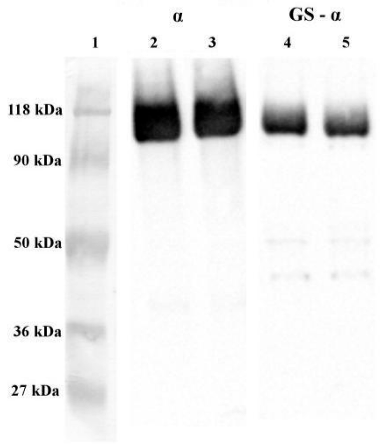

Figure S2. Deglutathionylation of the Na,K-ATPase α-subunit using the coupled enzyme system glutaredoxin (Grx) (20.6 μg/mL) and glutathione reductase (GR) in the presence of 0.5 mM glutathione (GSH) and 200 μM nicotinamide adenine dinucleotide phosphate (NADPH). Samples were incubated for 30 min at 37 °C. Control samples (taken as 100%) were incubated under the same conditions without reduced glutathione and the enzymes. The figure presents results of immunoblotting with staining by antibodies against bound glutathione and against Na,K-ATPase α1-subunit. 1—molecular weight standards; 2—control (anti-α1 antibody); 3—Grx + GR system (anti-α1 antibody); 4—control (anti-glutathione antibody); 5—Grx+GR system (anti-GSH antibody).

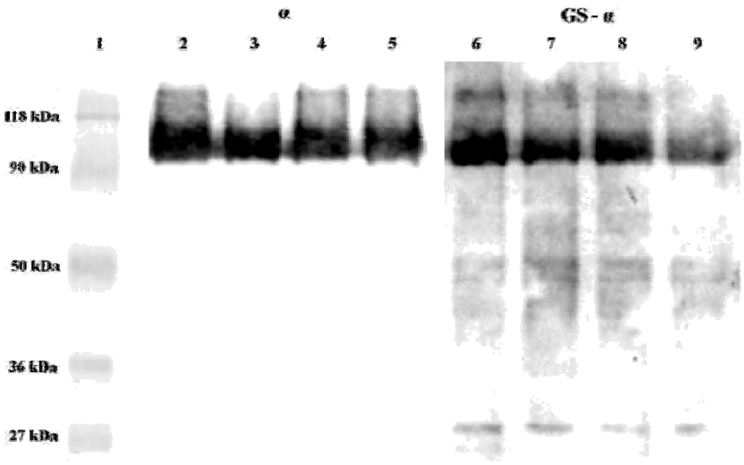

Figure S3. Deglutathionylation using chemical reducing agents: 25 mM tris(2-carboxyethyl)-phosphine (TCEP), 10 mM $Na_2S_2O_4$, and 3% $NaBH_4$. The incubation time was 30 min at 37 °C. The Figure depicts immunoblotting with staining by antibodies against bound glutathione and against the α1-subunit. The results were normalized to the content of the α1-subunit. 1—molecular weight standards; 2—control (anti-α1 antibody); 3—25 mM TCEP (anti-α1 antibody); 4—10 mM $Na_2S_2O_4$ (anti-α1 antibody); 5—3% $NaBH_4$ (anti-α1 antibody); 6—control (anti-GSH antibody); 7—25 mM TCEP (anti-GSH antibody); 8—10 mM $Na_2S_2O_4$ (anti-GSH antibody); 9—3% $NaBH_4$ (anti-GSH antibody).

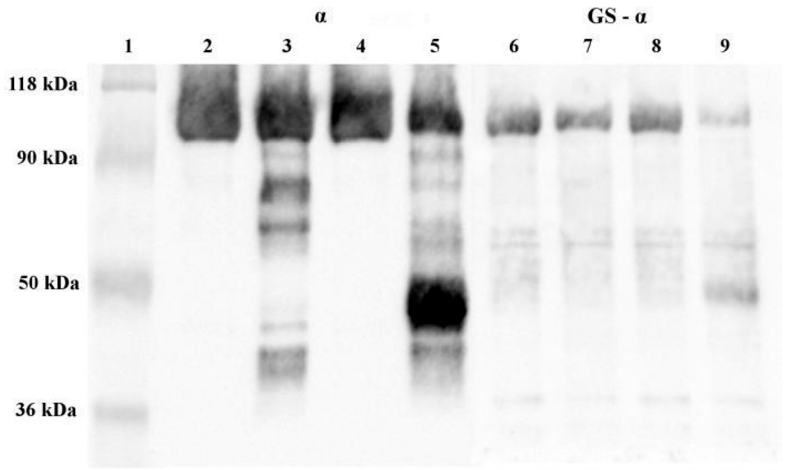

Figure S4. Deglutathionylation of duck salt gland α-subunit (after treatment with 25 mM TCEP, 10 mM $Na_2S_2O_4$, or 3% $NaBH_4$) in the presence of 8 M urea and 8% SDS for 30 min at 37 °C of immunoblotting after incubation of the enzyme with antibodies against bound glutathione and the α1-subunit are presented. The results were normalized to the content of the α1-subunit. 1—molecular weight standards; 2— control (anti-α1 antibody); 3— 25 mM TCEP (anti-α1 antibody); 4—10 mM $Na_2S_2O_4$ (anti-α1 antibody); 5—3% $NaBH_4$ (anti-α1 antibody); 6—control (anti-GSH antibody); 7—25 mM TCEP (anti-GSH antibody); 8—10 mM $Na_2S_2O_4$ (anti-GSH antibody); 9—3% $NaBH_4$ (anti-GSH antibody).

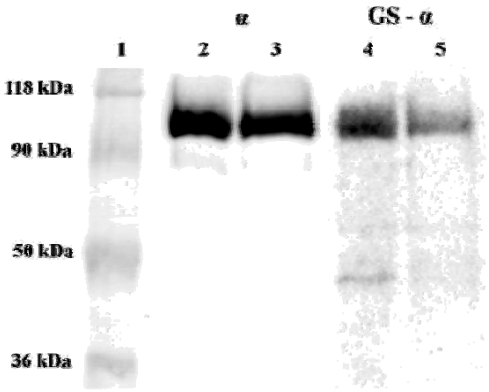

Figure S5. Microsomes of duck salt glands after treatment with 3% $NaBH_4$ in the presence of 8 M urea and 8% SDS for 30 min at 37 °C. Results of immunoblotting after incubation of the sample with antibodies against glutathione and the α1-subunit are presented. The results were normalized to the content of the α1-subunit. 1—molecular weight standards; 2—control (anti-α1 antibody); 3—3% $NaBH_4$ (anti-α1 antibody); 4—control (anti-GSH antibody); 5—3% $NaBH_4$ (anti-GSH antibody).

A B

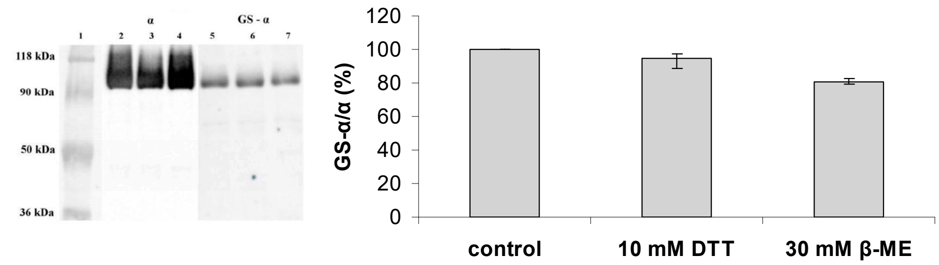

Figure S6. Deglutathionylation of the Na,K-ATPase α-subunit using chemical reducing agents: 10 mM DTT and 30 mM β-ME (β-mercaptoethanol). Samples were incubated for 30 min at 37 °C. Control samples (taken as 100%) were incubated under the same conditions without reducing reagents. (**A**) Original immunoblotting readouts; (**B**) results of immunoblotting analysis. The figure presents results of immunoblotting with staining by antibodies against bound glutathione and against Na,K-ATPase α1-subunit. 1—molecular weight standards; 2—control (anti-α1 antibody); 3—10 mM DTT (anti-α1 antibody); 4—30 mM β-ME (anti-α1 antibody); 5—control (anti-GSH antibody); 6—10 mM DTT (anti-GSH antibody); 7—30 mM β-ME (anti-GSH antibody) (number of experiments: N = 4).

A B

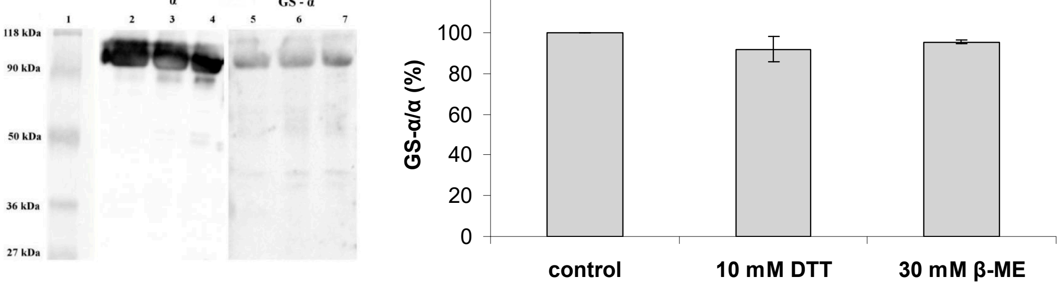

Figure S7. Deglutathionylation of the Na,K-ATPase α-subunit using chemical reducing agents 10 mM DTT and 30 mM β-ME (β-mercaptoethanol) in the presence of 8 M urea and 8% SDS for 30 min at 37 °C. (**A**) Original immunoblotting readouts; (**B**) results of immunoblotting analysis. The figure presents results of immunoblotting with staining by antibodies against bound

glutathione and the α1-subunit. The results were normalized to the content of the α1-subunit. 1—molecular weight standards; 2— control (anti-α1 antibody); 3— 10 mM DTT (anti-α1 antibody); 4—30 mM β-ME (anti-α1 antibody); 5—control (anti-GSH antibody); 6—10 mM DTT (anti-GSH antibody); 7—30 mM β-ME (anti-GSH antibody) (number of experiments: $N = 3$).

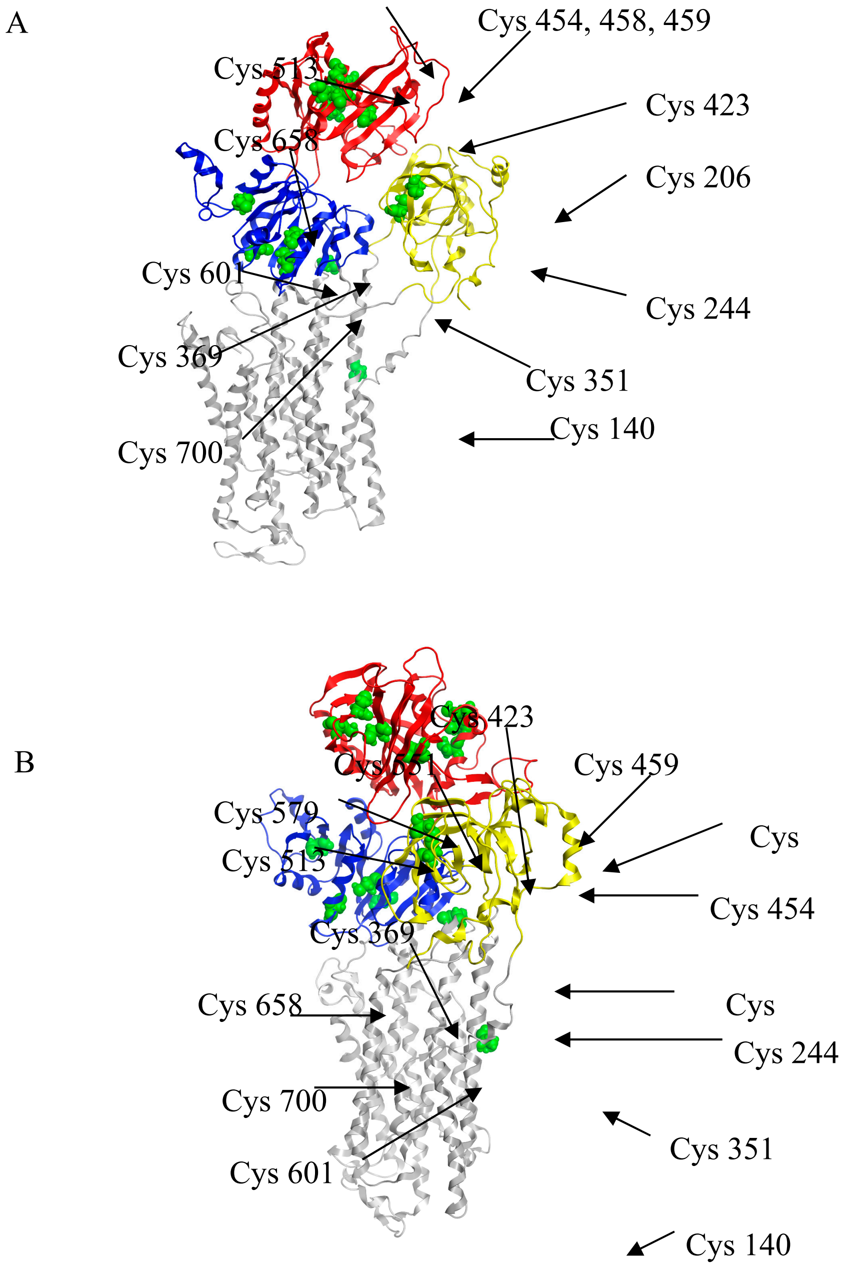

Figure S8. Cartoon representation of the Na,K-ATPase α1-subunit model in two projections (**A** and **B**), built on the basis of 2.8 Å structure of the porcine α1-subunit (Protein Data Bank code: 3wgu). Color coding of the Na,K-ATPase refers to individual domains, with the actuator (A) domain (residues 1 to 77 and 149 to 270) in yellow, the phosphorylated (P) domain (residues 363 to 376 and 589 to 753) in blue, the nucleotide binding (N) domain (residues 377 to 588) in red, and the transmembrane region (M1–M10) in grey. The cysteine residues' corresponding to the cysteine residues in Table 1 are shown as ball-and-stick representation in green. Numbering of cysteine residues corresponds to duck α1-subunit sequence. This figure was prepared with the MOE version 2013.08 modeling software (Chemical Computing Group Inc., Montreal, Canada).

Table S1. Cys-containing peptides (including sequences with different number of trypsin miscleavage sites) detected in matrix assisted laser desorption ionization-time of flight mass spectrometry (MALDI-TOF MS) spectra of trypsinized Na,K-ATPase α1-subunit.

Cysteine Residue No.	Fragment	Sequence	Experimental *m/z*	Calculated *m/z*	Cysteine Modification
140	123-148	DNLYLGIVLAAVVIITGCFSYYQEAK	3,168,742	3,168,563	SG
206	200-207	IISAHGCK	1,133,508	1,133,384	SG
206	200-207	IISAHGCK	82,844	828,349	SH
206	200-222	IISAHGCKVDNSSLTGESEPQTR	2,429,173	2,428,803	SH
244	223-250	SPDFSNENPLETRNIAFFSTNCVEGTAR	3,132,433	313,183	SOH
244	223-250	SPDFSNENPLETRNIAFFSTNCVEGTAR	3,116,438	3,115,842	SH
244	236-250	NIAFFSTNCVEGTAR	1,661,759	1,661,531	SO_2H
244	236-250	NIAFFSTNCVEGTAR	1,629,769	1,629,557	SH
351	346-354	MARKNCLVK	1,091,581	1,091,431	SNO
351	346-354	MARKNCLVK	1,062,591	1,062,407	SH
351	349-372	KNCLVKNLEAVETLGSTSTICSDK	2,874,353	2,874,865	SOH, SG
351	349-372	KNCLVKNLEAVETLGSTSTICSDK	2,890,348	2,889,819	SG, SO_2H
351	349-354	KNCLVK	736,402	736,271	SO_2H
351	350-372	NCLVKNLEAVETLGSTSTICSDK	2,486,175	2,485,812	SO_2H, SNO
369	355-372	NLEAVETLGSTSTICSDK	1,867,895	1,867,631	SH
369	360-385	NLEAVETLGSTSTICSDKTGTLTQNR	2,739,347	2,738,916	SH
423	419-425	VAGLCNR	732,382	732,304	SH
454, 458, 459	440-463	RAVAGDASESALLKCIELCCGSVK	2,455,199	2,454,809	SO_2H
454, 458, 459	441-463	AVAGDASESALLKCIELCCGSVK	2,331,088	2,330,795	SO_2H, SO_2H
454, 458, 459	454-466	CIELCCGSVKEMR	1,388,531	1,388,496	SG, SNO
454, 458, 459	454-466	CIELCCGSVKEMR	182,071	1,820,604	SG, SNO, SOH
454, 458, 459	454-468	CIELCCGSVKEMRER	185,177	185,166	SO_2H, SO_2H, SO_2H
454, 458, 459	459-468	CIELCCGSVK	1,388,531	1,388,496	SG, SNO
454, 458, 459	459-471	CIELCCGSVKEMR	1,836,705	1,836,759	SO_2H, SNO, SG
454, 458, 459	459-473	CIELCCGSVKEMRER	1,829,776	1,829,687	SNO, SOH, SNO

Table S2. Cys-containing peptides (including sequences with different number of trypsin miscleavage sites) detected in MALDI-TOF MS spectra of trypsinized Na,K-ATPase α1-subunit after reduction with sodium borohydride.

Cysteine Residue No.	Fragment	Sequence	Experimental *m/z*	Calculated *m/z*	Cysteine Modification
140	123-151	DNLYLGIVLAAVVIITGCFSYYQEAKSSK	3,194,644	3,194,998	SNO
206	194-207	IPADLRIISAHGCK	1,493,826	1,493,614	SH
206	200-222	IISAHGCKVDNSSLTGESEPQTR	2,429,173	242,899	SH
244	223-250	SPDFSNENPLETRNIAFFSTNCVEGTAR	3,116,023	3,116,438	SH
244	236-250	NIAFFSNCVEGTAR	1,661,759	1,661,628	SO_2H
244	236-250	NIAFFSTNCVEGTAR	1,629,648	1,629,769	SH
351	346-354	MARNCLVK	1,091,581	1,091,475	SNO
351	346-354	MARNCLVK	1,062,591	1,062,497	SH
369	355-372	NLEAVETLGSTSTICSDK	1,867,895	1,867,719	SH
369	360-385	NLEAVETLGSTSTICSDKTGTLTQNR	2,739,347	273,905	SH
423	419-425	VAGLCNR	732,382	732,343	SH
454, 458, 459	440-463	RAVAGDASESALLKCIELCCGSVK	2,486,996	2,487,189	SO_2H, SO_2H
454, 458, 459	440-463	RAVAGDASESALLKCIELCCGSVK	2,455,199	2,454,963	SO_2H
454, 458, 459	441-463	AVAGDASESALLKCIELCCGSVK	2,877,244	2,877,008	SG, SG
454, 458, 459	441-463	AVAGDASESALLKCIELCCGSVK	2,331,088	2,330,923	SO_2H, SO_2H
454, 458, 459	459-468	CIELCCGSVK	1,388,531	1,388,615	SG, SNO
454, 458, 459	459-473	CIELCCGSVKEMRER	1,829,776	1,829,786	SNO, SOH, SNO
454, 458, 459	459-468	CIELCCGSVK	1,118,453	1,118,474	SO_2H, SO_2H
454, 458, 459	459-468	CIELCCGSVK	1,086,463	1,086,501	SO_2H
454, 458, 459	459-468	CIELCCGSVK	1,102,458	1,102,533	SOH, SO_2H
454, 458, 459	459-468	CIELCCGSVK	1,054,473	1,054,443	SH, SH, SH
454, 458, 459	454-466	CIELCCGSVKEMR	1,557,628	1,557,607	SNO, SNO, SNO
513	509-530	ILDRCSSILLHGKEQPLDEEMK	2,859,369	2,859,069	SG
513	509-521	ILDRCSSILLHGK	1,454,815	145,469	SH
513	513-521	CSSILLHGK	1,262,587	126,255	SG
513	513-521	CSSILLHGK	957,519	957,467	SH
601	594-602	AAVPDAVGKCR	1,102,567	1,102,533	SOH
601	592-607	AAVPDAVGKCRSAGIK	1,558,837	1,559,116	SOH
